# Occipital GABA correlates with cognitive failures in daily life^☆^

**DOI:** 10.1016/j.neuroimage.2013.10.059

**Published:** 2014-02-15

**Authors:** Kristian Sandberg, Jakob Udby Blicher, Mia Yuan Dong, Geraint Rees, Jamie Near, Ryota Kanai

**Affiliations:** a Cognitive Neuroscience Research Unit, Aarhus University Hospital, Noerrebrogade 44, Building 10G, 8000 Aarhus C, Denmark; b UCL Institute of Cognitive Neuroscience, University College London, 17 Queen Square, WC1N 3AR London, UK; c Hammel Neurorehabilitation and Research Centre, Aarhus University Hospital, Voldbyvej 15, 8540 Hammel, Denmark; d Center for Functionally Integrative Neuroscience, Aarhus University Hospital, Noerrebrogade 44, Building 10G, 8000 Aarhus C, Denmark; e Wellcome Trust Centre for Neuroimaging, Institute of Neurology, 12 Queen Square, WC1N 3AR London, UK; f Douglas Mental Health University Institute and Department of Psychiatry, McGill University, 1033 Pine Avenue West, Montreal, QC H3A 1A1, Canada; g FMRIB Centre, University of Oxford, OxfordOX3 9DU, UK; h School of Psychology, Sackler Centre for Consciousness Science, University of Sussex, Falmer, BN1 9QH, UK

**Keywords:** Cognitive failures, CFQ, Gamma aminobutyric acid, GABA, Visual cortex, Superior parietal lobule

## Abstract

The brain has limited capacity, and so selective attention enhances relevant incoming information while suppressing irrelevant information. This process is not always successful, and the frequency of such cognitive failures varies to a large extent between individuals. Here we hypothesised that individual differences in cognitive failures might be reflected in inhibitory processing in the sensory cortex. To test this hypothesis, we measured GABA in human visual cortex using MR spectroscopy and found a negative correlation between occipital GABA (GABA +/Cr ratio) and cognitive failures as measured by an established cognitive failures questionnaire (CFQ). For a second site in parietal cortex, no correlation between CFQ score and GABA +/Cr ratio was found, thus establishing the regional specificity of the link between occipital GABA and cognitive failures. We further found that grey matter volume in the left superior parietal lobule (SPL) correlated with cognitive failures independently from the impact of occipital GABA and together, occipital GABA and SPL grey matter volume statistically explained around 50% of the individual variability in daily cognitive failures. We speculate that the amount of GABA in sensory areas may reflect the potential capacity to selectively suppress irrelevant information already at the sensory level, or alternatively that GABA influences the specificity of neural representations in visual cortex thus improving the effectiveness of successful attentional modulation.

## Highlights

• GABA/Cr ratio was measured for an occipital and a parietal voxel.
• Cognitive failures were measured using the cognitive failures questionnaire (CFQ).
• Cognitive failures in daily life correlate with occipital GABA/Cr ratio only.
• Parietal (lSPL) grey matter volume also correlated with cognitive failures.
• Together, the two factors statistically explain 51% of individual CFQ variability.

## Introduction

In everyday life, we continuously receive large amounts of information, some of which are relevant to our current goals and some of which are not. As the brain has limited capacity, attention allows relevant incoming information to be selectively enhanced while suppressing irrelevant information (Desimone and Duncan, 1995). However, cognitive failures occur every now and then: we often fail to listen to the name of a person we are being introduced to or miss an item we are looking for even though the item is within our visual field.

Such cognitive failures have been studied in psychology (Broadbent et al., 1982, Herrman and Neisser, 1978, Reason, 1977) and research using the cognitive failures questionnaire (CFQ) (Broadbent et al., 1982) has established a link between cognitive failures and selective attention (Forster and Lavie, 2007, Kanai et al., 2011b, Tipper and Baylis, 1987). Desimone and Duncan (Desimone, 1998, Desimone and Duncan, 1995) and Corbetta and Shulman (2002) propose that bottom-up attention should be conceptualised as a bias of stimulus saliency upon the competition between different representations of stimuli in extrastriate areas. In contrast, they propose that top-down attention represents an influence of prefrontal/parietal areas upon this competition, resulting in suppression of the neuronal representations of the behaviourally least relevant stimuli. Indeed, frontal and parietal sites are activated when a salient distractor is present in visual search tasks (De Fockert and Theeuwes, 2012, De Fockert et al., 2004). The link between grey matter (GM) volume (Kanai et al., 2011b) in the frontal and in the parietal cortex or the connection strength between these areas (Edin et al., 2007) and one component of cognitive failures as measured by the CFQ, namely distractibility, has recently been studied. However, so far our knowledge of the link between the characteristics of the sensory cortex and cognitive failures is very limited.

The inhibitory neurotransmitter gamma-aminobutyric acid (GABA) plays a central role in determining visual cortex selectivity. Pharmacological studies in cats show that GABA mediates stimulus selectivity in the visual cortex (Iversen et al., 1971, Sillito, 1975, Sillito, 1979, Tsumoto et al., 1979), and GABA is involved in interocular suppression between cells in the visual cortex (Sengpiel and Vorobyov, 2005). Furthermore, a recent study in humans showed that visual cortex GABA (measured by MR spectroscopy) correlates positively with suppression duration during ambiguous perception and that Lorazepam (a GABA_A_ agonist) increases suppression duration (van Loon et al., 2013). This study indicates that the visual cortex GABA not only correlates with the degree of suppression of a competing representation but also has a causal impact upon it. Finally, studies of individuals with attention-deficit/hyperactivity disorder (ADHD) point to a link between sensory cortex GABA and cognitive failures, as these individuals exhibit higher incidences of failures in behavioural inhibition (e.g. inattention) and have lower GABA concentration in the somatosensory and primary motor cortices compared to healthy controls (Edden et al., 2012, Tannock, 1998).

Overall, GABA thus plays an important part in stimulus processing and suppression in sensory areas. In the present study, we hypothesised that GABA levels in the occipital cortex would be correlated with self-reported cognitive failures as measured by CFQ scores. Or more generally: the more sensory GABA, the greater inhibitory capacity and the greater chance that overt, selective attention succeeds in suppressing distractors. We thus examined the correlation between visual cortex GABA concentration (as measured by MR spectroscopy) and the frequency of self-reported cognitive failures in daily life (as measured by the CFQ). Given the links between stimulus selectivity and cognitive failures as well as between stimulus selectivity and GABA, we hypothesised that higher the visual cortex GABA leads to fewer incidences of cognitive failures.

The sensory cortex is not the only area of the brain thought to be involved in distractor suppression. Although we are aware of no study examining the role of parietal GABA, much evidence points to the involvement of the parietal cortex in attentional selection, specifically the superior parietal lobule (SPL): Grey matter volume of both the posterior and the anterior right SPL correlate with the frequency of changes in the direction of suppression during ambiguous perception, and disruption of activity at these sites impacts on the frequency of changes of suppression direction (Carmel et al., 2010, Kanai et al., 2010, Kanai et al., 2011a). Additionally, as mentioned above, left SPL grey matter volume correlates with the distractibility component of the CFQ (Kanai et al., 2011b, p. 2). In order to test if the amount of GABA in SPL correlated with cognitive failures, a second spectroscopy voxel was placed here. To ensure that GABA spectroscopy results are not confounded by the overall neuronal density, it is often tested that there is no correlation between the measure of interest (here the CFQ) and grey matter volume within the spectroscopy voxel (cf. Sumner et al., 2010). As we predicted that left SPL grey matter volume correlated with CFQ score (cf. Kanai et al., 2011b), the parietal voxel was placed in the right SPL to avoid this confound.

## Materials and methods

### Participants

Only males participated in the experiment as cortical GABA concentration varies with the menstrual cycle in females (Harada et al., 2011). 36 healthy young males gave written informed consent to participate in the experiment. They were between 20 and 40 years of age (mean = 25.4, SD = 4.70) and had normal or corrected-to-normal vision. The experiments were approved by the local ethics committee, De Videnskabsetiske Komitéer for Region Midtjylland, Denmark.

### The cognitive failures questionnaire (CFQ)

All 36 participants filled out the CFQ. The CFQ measures cognitive failures in everyday life by asking participants to rate the frequency with which they experience 25 common cognitive failures in perception, memory, and motor function on a scale from 0 (never) to 4 (very often). An example of a question is “Do you fail to notice signposts on the road?”. Factor analysis of the CFQ has revealed four internally consistent, interpretable factors: memory, distractibility, blunders, and (memory for) names (Wallace et al., 2002). The total score obtained shows moderate correlations with reports of cognitive failures by spouses, with Herrmann and Neisser's (1978) memory questionnaire, with Reason's (1977) inventories of slips of action and of absentmindedness (Broadbent et al., 1982). Furthermore, the total CFQ score shows a moderate correlation with an experimental measure of distractibility, the attentional capture effect (Kanai et al., 2011b).

### Magnetic resonance imaging (MRI)

Participants were scanned on a Siemens Tim Trio 3 T MRI-scanner (Erlangen, Germany). A T1 MPRAGE structural scan (TR/TE 2420/3.7 ms, 1 mm isotropic resolution, scan time 5 1/2 min) was performed and used for subsequent voxel placement and voxel segmentation. GABA edited MRS was performed using MEGA-PRESS (Edden and Barker, 2007, Mescher et al., 1998). In brief, the methods acquire two different spectra; one with an editing pulse targeting the C3-GABA peak at 1.9 ppm (edit on) and another with the editing pulse symmetrically on the other side of the water peak (7.5 ppm, edit off). By subtracting the two spectra, the C4-GABA peak at 3 ppm (affected by the editing pulse through J-coupling with the C3-GABA protons) becomes visible and can be quantified. As a coupled macromolecule resonance at 3 ppm is also co-edited, and thus contributes to the measured signal, the term GABA + is often used. By adding the two spectra, instead of subtracting, the Creatine (Cr) peak at 3.0 ppm can be quantified and a GABA +/Cr ratio can be calculated.

**Fig. 1:**
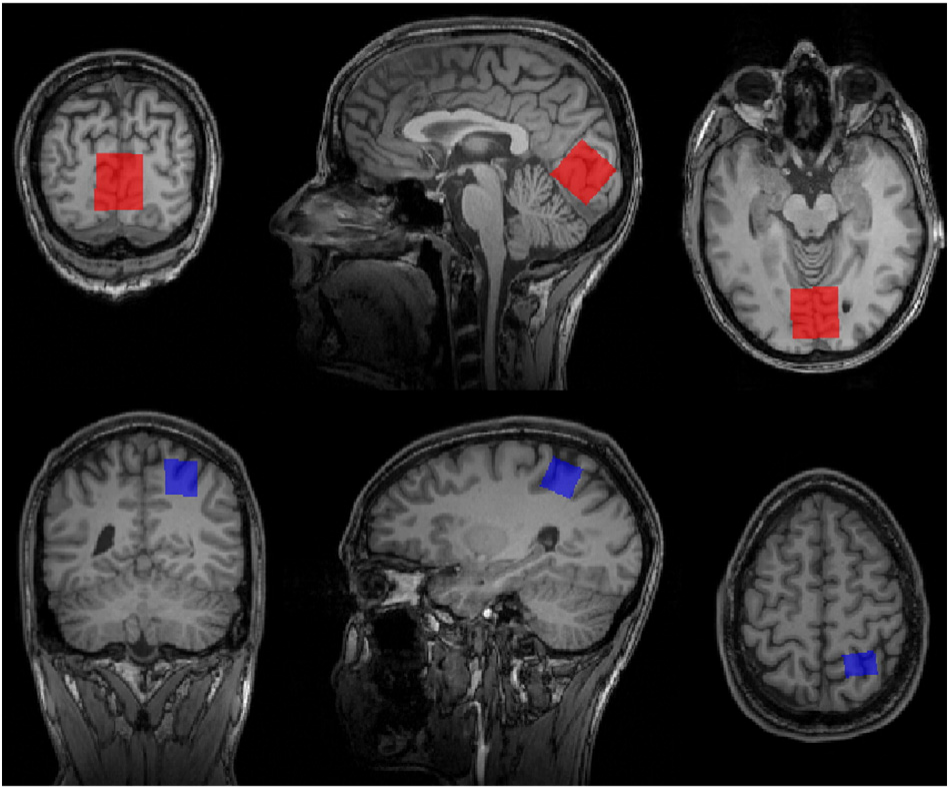
Voxel placement. Top: Placement of occipital MR spectroscopy voxel. The voxel covered the calcarine sulcus bilaterally. One edge was aligned with the parieto-occipital sulcus and then shifted as far towards the tentorium cerebelli and the occipital pole as possible without including the scalp and/or the tentorium cerebelli in the voxel. Bottom: Placement of parietal MR spectroscopy voxel. The right hand knob area was used to identify the precentral gyrus, which in turn was used to identify the postcentral gyrus. The voxel was placed so that the anterior border was parallel to the postcentral gyrus, thus centred on the anterior part of the superior parietal lobule.

For MEGA-PRESS MRS, the scan parameters were TR/TE: 2500/68 ms. For the occipital MRS, a 3 × 3 × 3 cm voxel was used and a total of 96 averages (edit on and edit off) were measured, leading to a scan time of 8 min. For the parietal MRS a 2 × 2 × 2 cm voxel was used and a total of 240 averages were measured, leading to a scan time of 20 min. For the occipital voxel (Fig. 1, top), the calcarine sulcus and the parieto-occipital sulcus were identified bilaterally. The voxel was placed so that it covered the calcarine sulcus bilaterally and so that one edge was aligned with the parieto-occipital sulcus and then shifted as far towards the tentorium cerebelli and the occipital pole as possible. Care was taken to avoid including the scalp and/or the tentorium cerebelli in the voxel. For the parietal voxel (Fig. 1, bottom), the right hand knob area on the precentral gyrus was identified (Yousry et al., 1997), and the voxel was placed so that the anterior border was parallel to the postcentral gyrus, thus centred on the anterior part of the superior parietal lobule. Care was taken to avoid including the scalp. The order in which the spectroscopy voxels were scanned was randomised between individuals.

GABA levels do not change depending of the time of the day (Evans et al., 2010), and they remain stable across at least a week for males (Harada et al., 2011). For these reasons, the time of the day for the MRS scan was not controlled for between participants, but participants completed the cognitive failures questionnaire within one week of the MRS scan.

### Analysis

#### Magnetic resonance spectroscopy

MRS data were analysed by author JUB who was blind to the CFQ scores. Custom MATLAB (Natick, MA) scripts were used for the removal of motion corrupted averages, drift correction and phasing of individual MRS data. Subsequently, the AMARES package (Vanhamme et al., 2001) within jMRUI (Naressi et al., 2001) software was used to estimate GABA + from the difference spectra and Creatine from the summed spectra. The final results were expressed as the GABA +/Cr ratios.

A visual data quality check was performed in combination with objective quality criteria. Data were excluded if they showed line broadening (line width > 8 Hz) or had high fit uncertainty of the Creatine peak in AMARES (SD/amplitude ratio > 0.20). 7 of the 72 dataset were excluded (3 occipital and 4 parietal) due to either misplacement of voxel during scan (one parietal), line broadening (one parietal and one occipital) or fit uncertainty caused by data being contaminated by signal from lipids (voxels placed too close to the scalp) (two parietal and two occipital). For the remaining datasets, both line width and SD/amplitude ratio were similar for the occipital (mean line width of 5.4 Hz and SD/amplitude of 0.03) and the parietal (mean line width of 4.8 Hz and SD/amplitude of 0.04) datasets.

Signal-to-noise ratio (SNR) was calculated using the difference spectrum following phase adjustment such that the NAA peak was upright with a phase of 0 degrees. Signal was calculated as the maximum intensity of the real part of the NAA peak in the phased difference spectrum, and noise was calculated as the standard deviation of the real part of the noise in a signal-free part of the spectrum following a baseline correction to remove any 1st and 2nd order baseline variations. Although a higher SNR was obtained for the occipital voxel (226) than for the parietal voxel (108), both SNRs were acceptably high for the detection of GABA.

#### Segmentation

To test whether a correlation between measured GABA +/Cr ratio and CFQ could be explained simply by differences in the overall structural composition within the spectroscopy voxels we performed a segmentation of voxel content into grey matter (GM), white matter (WM) and cerebrospinal fluid (CSF). Segmentation was performed using the standard segmentation tool within the Statistical Parametric Mapping software (SPM8, http://www.fil.ion.ucl.ac.uk/spm/). Using the DICOM of the voxel location as a mask, the volume of each tissue type (GM, WM, CSF) was calculated for the occipital and parietal voxels. Tests for correlation between CFQ and tissue types were then performed.

In addition, we performed a voxel-based morphometry (VBM) analysis using the T1 MPRAGE structural scans collected from the same participants. The purpose of this analysis was to examine whether occipital GABA predicted cognitive failures independently of lSPL GM volume (Kanai et al., 2011b). The VBM analysis was performed following exactly the same procedure as the original study (see Kanai et al., 2011a, Kanai et al., 2011b for details). The analysis was focused on the small volume defined as a sphere (10 mm radius) centred at the peak coordinate in the previous study (MNI, x = − 15, y = − 61, z = 54). We used p < 0.05 corrected for the small volume as defined above as the criterion for statistical significance. For this analysis, the distractibility subscale derived from Wallace et al. (2002) was used in the main analysis instead of the total CFQ score, as in the previous study, but the independence of the impact of GABA and lSPL GM volume on total CFQ was also tested.

### Statistics

Data were analysed using the Pearson product–moment correlation, which has five assumptions: 1) That the variables are interval or ratio measurements, 2) that variables are approximately normally distributed, 3) that there is a linear relationship between the two variables, 4) that outliers are kept to a minimum or removed entirely, and 5) that there is homoscedasticity of the data.

All these assumptions were tested for the variables CFQ score, occipital and parietal GABA +/Cr ratio, as well as for grey matter volume, white matter volume, and cerebrospinal fluid volume of the occipital and parietal GABA voxels, and for grey matter volume of the lSPL site. All variables were interval measurements. Shapiro–Wilk tests and histogram inspection did not refute the assumption of normality for any variable (W > 0.96, p > 0.24 for all variables). All relationships were linear (see the Results section). No outliers were observed. Cook–Weisberg tests failed to refute the assumption of homoscedasticity (chi^2^(1) < 2.06, p > 0.15 for all main analysis variables (occipital and parietal GABA +/Cr ratio and lSPL GM volume) and chi^2^(1) < 3.33, p > 0.06 for all control analysis variables).

## Results

### Correlation between occipital GABA and cognitive failures

**Fig. 2:**
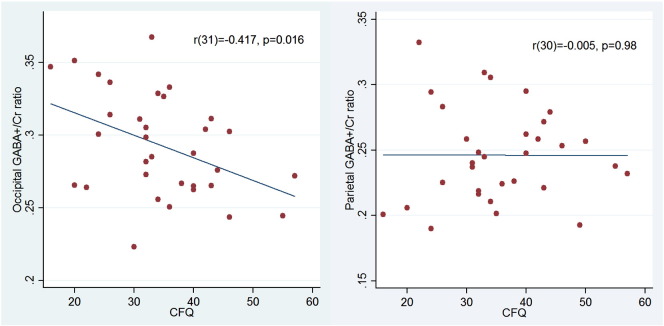
Correlation between CFQ score and GABA +/Cr ratio. CFQ score and GABA +/Cr ratio is plotted for all participants (one point representing one participant). Linear regression lines are fitted for both plots and the results of a Pearson product–moment correlation analysis (CFQ vs. GABA +/Cr) is reported in the top right corner of each plot. Left: CFQ vs. occipital GABA +/Cr ratio. Right: CFQ vs. parietal (right aSPL) GABA +/Cr ratio. Note that a negative correlation was found between occipital GABA +/Cr and cognitive failures whereas no correlation was found between aSPL GABA +/Cr and cognitive failures.

There was a significant negative correlation between CFQ score and occipital GABA +/Cr ratio: r(31) = − 0.417, p = 0.032 (Bonferroni corrected for multiple comparisons) (Fig. 2). GABA +/Cr ratio correlated best with the blunders factor (r(31) = − 0.412, p = 0.017), memory factor (r(31) = − 0.385, p = 0.027) and distractibility factor (r(31) = − 0.318, p = 0.072), but not significantly with the name factor (r(31) = − 0.131, p = 0.47). The overall CFQ correlation was selective to the visual cortex as no such correlation was found for the voxel located in the right anterior SPL — the correlation between GABA +/Cr at this voxel and CFQ score was: r(30) = − 0.005, p = 0.98. Finally, the correlation could not be explained by the amount of grey matter, white matter, or cerebrospinal fluid in the occipital GABA voxel for individuals with fewer cognitive failures, as no correlation was found between CFQ scores and any of these variables: |r(34)| < 0.06, p > 0.75, for all comparisons. The corresponding correlations for the parietal voxel were |r(34)| < 0.16, p > 0.36, for all comparisons.

### Correlation between parietal GM volume and cognitive failures

**Fig. 3:**
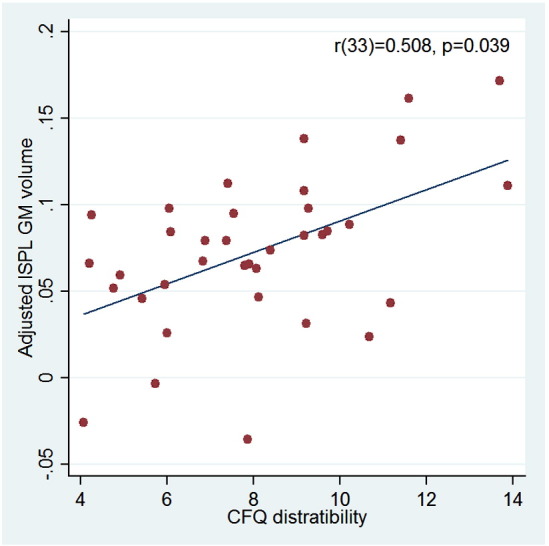
Replication of correlation between CFQ distractibility score and adjusted lSPL GM volume (Kanai et al., 2011b). CFQ distractibility score and adjusted lSPL GM volume is plotted for all participants (one point representing one participant). Linear regression lines are fitted and the results of a Pearson product–moment correlation analysis (distractibility vs. GM volume) are reported in the top right corner. No significant relationship was found between adjusted lSPL GM volume and occipital GABA/Cr ratio, thus indicating that occipital GABA and SPL GM volume contribute independently to the occurrence of cognitive failures.

As mentioned above, Kanai et al., 2011a, Kanai et al., 2011b reported a positive correlation between GM volume in the left SPL and the distractibility component of the CFQ. We were interested in examining if occipital GABA +/Cr ratio and lSPL grey matter volume were independent predictors of cognitive failures. First, we replicated the finding of Kanai et al. (2011b) in the present study by correlating the regional GM volume within a sphere (r = 10 mm) around the MNI coordinates (MNI, x = − 15, y = − 61, z = 54) with the distractibility score (Fig. 3). Within the sphere, the peak correlation was found at x = − 20, y = − 61, z = 54, and the correlation coefficient R = 0.508 (t(33) = 3.3832, p = 0.039, corrected for small volume). Having replicated the effect, we examined whether it was independent of any GABA-related correlation by testing the correlation between the GM volume at the peak voxel in the sphere and occipital GABA +/Cr ratio. No significant relationship was found (R = 0.209, p = 0.24, one-tailed), thus indicating that occipital GABA and SPL grey matter volume contributed independently to the occurrence of cognitive failures. In support of this notion, the correlation between occipital GABA +/Cr ratio and CFQ score remained significant even after regressing out the influence of SPL GM (R = − 0.605, p < 0.001). The correlation between lSPL grey matter volume and the overall CFQ score was r(33) = 0.413, p = 0.012 (uncorrected). Finally, we tested the impact of occipital GABA +/Cr ratio and lSPL grey matter volume on CFQ score in one multiple linear regression model. This model showed that, together, the two variables predicted 50.9% of the CFQ score (F(2,30) = 15.5, p < 0.0001) and, more importantly, that both GABA (t(33 = − 4.00, p < 0.001) and lSPL grey matter volume (t(33 = 4.53, p < 0.001) contributed significantly to the predictability of the model.

## Discussion

In the present study, we found that occipital GABA was correlated with the individual differences in self-reported cognitive failures in daily life. We demonstrated that the results could not be accounted for by grey/white matter volume or cerebrospinal fluid volume in the occipital target for GABA spectroscopy as no significant correlation between CFQ score and any of these measures was found. Furthermore, we demonstrated that the results could not be caused by less distractible individuals having generally high GABA levels in their attentional network as no correlation was found between CFQ score and GABA +/Cr ratio of the voxel in the right anterior SPL. This result fits well with theories of visual attention, which posit that top-down attention is related to frontal/parietal areas whereas bottom-up attention is related to mutually suppressive signals in sensory areas (Desimone, 1998, Desimone and Duncan, 1995). Here, we thus demonstrated that the neurochemical characteristics of the visual cortex statistically explain part of the individual variability in the prevalence of cognitive failures, which are linked to selective attention.

Furthermore, we replicated a previous finding that left SPL grey matter volume correlated with one component of cognitive failures: distractibility (Kanai et al., 2011b). Analyses revealed that, together, left SPL GM volume and visual GABA +/Cr ratio statistically explained 51% of the individual variation in CFQ score and that the two factors were best described as independent predictors of cognitive failures/distractibility. This is interesting as it means that two individuals experiencing the same number of cognitive failures may have different neural origins if the results can be interpreted causally.

Of the four factors of cognitive failures, lSPL grey matter volume predicted distractibility the best whereas GABA +/Cr ratio predicted the blunders and memory factors the best but also distractibility to a relatively high degree. These slightly different profiles may be taken as further support that lSPL grey matter volume and occipital GABA +/Cr ratio explain slightly different, yet overlapping, aspects of cognitive failures.

It should be noted that we base our conclusions on the observation of correlations, which does not necessarily imply causality. It is possible that some yet unknown mechanism is responsible for both visual cortex GABA concentration and cognitive failures in an independent manner. In order to establish support for a causal relationship between GABA and cognitive failures, future studies would need to manipulate GABA concentration. GABA concentration can be modulated either pharmacologically using, for instance, Vigabatrin (Weber et al., 1999) or indirectly using transcranial direct current stimulation (tDCS) (Stagg et al., 2009). Such intervention methods will be required to establish causal role of occipital GABA concentration in cognitive failures.

It should further be noted that the absence of a correlation between the CFQ score and right anterior parietal GABA +/Cr ratio should not be taken as evidence that GABA has no role in parietal lobe processing; the results are specific to the relation between GABA in the right anterior parietal lobe and cognitive failures. Additionally, it should be noted that the null finding for parietal GABA in principle could be caused by other factors than the absence of a relationship. Specifically, we cannot rule out with complete certainty that the failure to find a significant relationship in the parietal region was due to an approximately two-fold lower SNR in the parietal voxel than in the occipital voxel (despite the prolonged scan time for the parietal voxel to account for the smaller volume). However, we judge this possibility to be unlikely, given that the SNR in the parietal region, although lower than in the occipital region, was acceptably high for the detection of GABA, and yet we still did not observe even the slightest trend towards significance in this region (R = − 0.005, p = 0.98). Furthermore, similar line widths and SD/amplitude ratios were observed for the two voxels.

As mentioned above, GABA MRS measures the amount of GABA (in relation to, for instance, Creatine), but it is still unknown whether the measured transmitter level reflects phasic or tonic inhibition or a combination of both (Stagg et al., 2011). Further studies combining GABA MRS and paired pulse transcranial magnetic stimulation (TMS) (Kujirai et al., 1993) or flumazenil PET (Qin et al., 2012) could help clarify the underlying physiology through which occipital GABA may impact causally on cognitive failures, as both techniques are sensitive to GABA-a receptor changes.

The results of our experiment also suggest that studies in other sensory modalities may be worthwhile. Auditory stimuli are often distracters in daily life, and we hypothesise that there will be a correlation between auditory cortex GABA concentration and cognitive failures. Finally, since sensorimotor cortex GABA concentration correlates with tactile discrimination ability (Puts et al., 2011) and since the CFQ also measures cognitive failures in the motor domain (e.g. “Do you bump into people?”), GABA concentration in motor cortex may also predict cognitive failures.

A previous study (Boy et al., 2010) observed a negative correlation between GABA concentration in the supplementary motor area and response priming effect size, i.e. the ability to act upon an *unperceived* but behaviourally relevant cue. This is somewhat different from our study in which participants with high occipital GABA levels report being better able to ignore irrelevant information. If the relevant information were also inhibited, we would not expect that these individuals would experience fewer cognitive failures. The subliminal nature of the stimulus used by Boy and colleagues prevents the participants from intentionally modulating attention. In contrast, when we perform goal-directed behaviour in our everyday lives, we are by definition aware of our goal, and we are expected to have a good idea of which information to enhance and which to suppress. We speculate that this difference may indicate mechanistically that a high GABA concentration does not simply lead to high levels of automatic, suppression of bottom-up signals (although this may also be the case), but more importantly that the inhibition can be selectively manipulated in a top-down manner to fit the current behavioural goals. This, again, fits well with theories of top-down modulation of attention and the finding of a negative correlation between distractibility and SPL GM volume by Kanai et al. (2011b). As such, occipital GABA levels may reflect the potential strength of suppression between competing visual stimuli, and top-down signals from frontal and parietal regions are more likely to be successful in their selection of a relevant target if irrelevant targets are effectively suppressed by the selected representation through lateral connections already at the sensory stage of processing.

Another, possibly complimentary, explanation is related to stimulus specificity, i.e. the distinctiveness of the neural representations of a stimulus. GABA levels have been associated with the selectivity or specificity of neural representations in both animals (Leventhal et al., 2003) and humans (Park et al., 2004). Such specificity mediates performance in a number of so-called visual fluid processing tasks (e.g. dot size comparison and symbol copying) (Park et al., 2010), and specificity may thus also have an impact on cognitive failures as it is expected that selective amplification or inhibition is better achieved in a system where stimuli are represented by distinct neuronal populations than in a system where there is large overlap in the neurons representing different objects.

One study reported a correlation between the GABA level of the frontal eye field (FEF) and the ability to resist conflict imposed by task irrelevant stimuli in a saccade task (Sumner et al., 2010). However, they did not find a correlation between occipital GABA and individual variability in this saccadic measure of distractibility. This apparent discrepancy with the present findings may be due to differences in the exact nature of the distractibility measure. In that earlier paper, distractibility was measured as the slowing of saccadic eye movements when a target was preceded by the sudden appearance of a salient irrelevant distractor. While competition between the distractor and saccade target could occur at multiple stages, the saccade task is likely to be dependent on conflict resolution within FEF (Schlag et al., 1998) rather than within early visual cortices. On the other hand, our measure of cognitive failures (the CFQ) encompasses a much wider range of inattention in everyday life. Our findings suggest that the link between the inhibitory capacity and attention control may not only be limited to regions associated with top-down attention, but may also be found in early sensory regions if a task is used in which sensory suppression is more critical.

In sum, the present study found that cognitive failures in daily life correlated with GABA +/Cr ratio in the occipital lobe, with GABA statistically explaining around 17% of the inter-individual variability. The results are correlational and further studies are needed to support any causal claims for a relationship between GABA and cognitive failures. One potential causal explanation could be that the inhibitory capacity in sensory areas affects our ability to ignore information that is irrelevant to our current behavioural goals. Another explanation could be that GABA causes increased specificity of visual representations and that this improves the chances of successful attentional modulation. We further demonstrated that the impact of SPL GM volume upon cognitive failure is independent of the impact of occipital GABA. Finally, we showed that our findings were not confounded by the overall cortical GABA levels or the structural composition of the target occipital area. These findings open up for further studies into the causal effect of GABA in cognitive failures or failures of attention in general, both as reported by participants in everyday lives, and in clinical and laboratory contexts.
